# Genetic analysis of rice seedling traits related to machine transplanting under different seeding densities

**DOI:** 10.1186/s12863-020-00952-1

**Published:** 2020-11-26

**Authors:** Dan Zhu, Yuping Zhang, Jing Xiang, Yaliang Wang, Defeng Zhu, Yikai Zhang, Huizhe Chen

**Affiliations:** State Key Laboratory of Rice Biology, China National Rice Research Institute, Hangzhou, China

**Keywords:** Rice seedling trait, Machine transplanting, Seeding density, QTL mapping, Additive and epistasis effect

## Abstract

**Background:**

Due to the diversity of rice varieties and cropping systems in China, the limitation of seeding density and seedling quality makes it hard to improve machine-transplanted efficiency. Previous studies have shown that indica and japonica varieties varied in machine transplanting efficiency and optimal seeding density. In this study, a RIL population derived from ‘9311’ and ‘Nipponbare’ were performed to explore the seedling traits variations and the genetic mechanism under three seeding densities.

**Results:**

The parents and RIL population exhibited similar trends as the seeding density increased, including seedling height and first leaf sheath length increases, shoot dry weight and root dry weight decreases. Among the 37 QTLs for six traits detected under the three seeding densities, 12 QTLs were detected in both three seeding densities. Five QTL hotspots identified clustered within genomic regions on chromosomes 1, 2, 4, 6 and 11. Specific QTLs such as *qRDW*_*1.1*_ and *qFLSL*_*5.1*_ were detected under low and high seeding densities, respectively. Detailed analysis the QTL regions identified under specific seeding densities revealed several candidate genes involved in phytohormones signals and abiotic stress responses. Whole-genome additive effects showed that ‘9311’ contributed more loci enhancing trait performances than ‘Nipponbare’, indicating ‘9311’ was more sensitive to the seeding density than ‘Nipponbare’. The prevalence of negative epistasis effects indicated that the complementary two-locus homozygotes may not have marginal advantages over the means of the two parental genotypes.

**Conclusions:**

Our results revealed the differences between indica rice and japonica rice seedling traits in response to seeding density. Several QTL hotspots involved in different traits and specific QTLs (such as *qRDW*_*1.1*_ and *qFLSL*_*5.1*_) in diverse seeding densities had been detected. Genome-wide additive and two-locus epistasis suggested a dynamic of the genetic control underlying different seeding densities. It was concluded that novel QTLs, additive and epistasis effects under specific seeding density would provide adequate information for rice seedling improvement during machine transplanting.

**Supplementary Information:**

The online version contains supplementary material available at 10.1186/s12863-020-00952-1.

## Background

Along with the social and economic development, rural labor transfer and ageing, traditional rice planting by hand technology has been unable meet the demands for rice production in China. Mechanization of rice cultivation is of great significance to improve rice production capacity and ensure national food security [1, 2]. Until 2012, the comprehensive mechanization level of rice production in China was 68.82%, of which the level of machine tillage and harvest were 93.29 and 73.35%, respectively. However, the level of machine transplanting only accounted for 31.67%, which was the bottleneck of rice production mechanization [3]. Although rice mechanical transplanting technology has been explored since the 1950s in China, there are still many problems on cropping system and varieties application. The rice mechanical transplanting technology including mechanical equipment and seedling raising technology widely applied in northern China was derived from Japan based on conventional japonica rice, which was restricted applied in indica varieties and hybrid rice with double season and multiple cropping system in southern China [4–9]. In particular, due to the tight growing season, the limitation of seeding density and seedling quality makes it more difficult to improve the efficiency for mechanized transplanting [10–13]. Although it has been used in hybrid rice, which accounts for about 60% of the rice producing area, the traditional rice mechanical transplanting technology cannot fully exploit the high yield advantage of hybrid rice, mainly due to the poor seedling cultivation, such as high seeding density, poor quality of seedlings, high rate of seedlings injury and large amount of seedlings per hill [14–17]. Therefore, due to the diversity of growth characteristics of different types of cultivars, it is necessary to make the seedlings with uniform size, consistent growth, and seedling characteristics suitable for appropriate ecological zones, planting systems and planting methods under machine transplanting.

The most important factor limiting the efficiency of machine transplanting was seedling cultivation, and the optimum rice seedlings were restricted by some factors including cultivation methods, nursery substrates, variety types and seeding density [8, 15, 18–20]. In general, it takes less time to recover from transplanting shock for young seedling with three to four leaves, seedling height ranging from 12 to 17 cm. Meanwhile, strong root cross-linked to stabilize the seedbed on the seedling-nursery tray not only reduced seedling damage during machine transplanting, but also conducive to the occurrence of tillers and the formation of yield [21]. Previous studies have shown that different rice varieties varied in machine transplanting efficiency and optimal seeding density [7, 20, 22]. Recently, the bowl-shaped blanket-like seedling transplanting technology combining the special machine transplanting seedling substrate, effectively solved the problems existing in the traditional blanket and potted seedling cultivation, including poor quality and quantitative positioning, high rate of seedling leakage and injury and reducing other adverse effects and the incidence of blight [1, 23, 24]. Seeding density was one of the most important constrains to produce good quality of rice seedlings in the seedling-nursery tray and were related to the characteristics of rice varieties [25]. Previous reports have suggested that seedlings with high density always have evenly emergence than those with the low density, and the consolidation force of the root system could be well formed into a blanket with the increase of seeding density. However, the quality of seedlings became worse with the increase of seeding density, and the seedling quality indexes such as dry matter of seedlings and root vigor showed a decreasing trend with the increase of seeding density [7, 16, 18, 20, 26]. Therefore, it is important to improve the efficiency of mechanical transplanting by selecting suitable seeding density for the special variety and raising method.

Many efforts have been made to improve seedling cultivation by providing plant growth regulators, which might cause several undesirable consequences such as seedling dwarfing, growth retardation and even reduction in yield if not used properly [27]. Therefore, it is urgent to investigate the genetic basis of seedling traits related to machine transplanting and improve the seedling quality through marker-assisted breeding. The essential problem of rice seedling response to seeding density was shade avoidance response, which referred to a set of architectural responses including accelerated growth of hypocotyl, internode, and petiole, decreased leaf surface area and chlorophyll and changed of leaf angle [28]. Competing for light and nutrients between adjacent plants at different seeding densities usually associated with balancing the investments in roots and leaves for rice seedlings. The molecular mechanisms of the hypocotyl, internode, or petiole elongation in shade avoidance response had been mainly characterized in dicots, depending on the cascade reaction of the light signal system, plant hormone signaling pathways, and growth regulation [29–31]. Previous studies showed that shading treatment through red to far-red light ratio (R/FR) could induce rice seedling stem elongation, which was contributed by phytohormones signals [32]. Beyond that, planting density may also affect rice root system growth through root-root recognition mediated by root exudates [33]. Identification of quantitative trait loci (QTLs) and candidate genes associated with early vigor such as mesocotyl elongation, shoot length, stem length, fresh weight, dry weight, and root vigor had been undertaken using different types of mapping population in rice under direct-seeding system [34–41]. Although young seedling traits associated with dry weight and root vigor had been extensively researched under controlled laboratory conditions [25, 42, 43], there have been very few reports on combining genetic research for early seedling traits related to mechanical transplanting directly on seedling nursery tray as well as controlled condition such as seeding density or temperature [44, 45]. The combined analysis would help in detecting QTLs that across different seeding densities as well as the special QTLs for certain traits that were important for mechanical transplanting seedling cultivation system. Therefore, the objectives of our research were applying a RIL population derived from indica and japonica cross: (1) to explore the trait performance across different seeding densities; (2) to compare the differences of QTLs for seedling traits under different seeding densities; (3) to analyze the whole-genome additive effects and epistatic effects under different seeding densities; (4) to detect novel loci controlling seedling traits under different seeding densities. These results would provide useful information for machine-transplanted rice seedling cultivation improvement in southern China rice-growing districts.

## Results

### Seedling trait performance of RILs and their parents

**Fig. 1:**
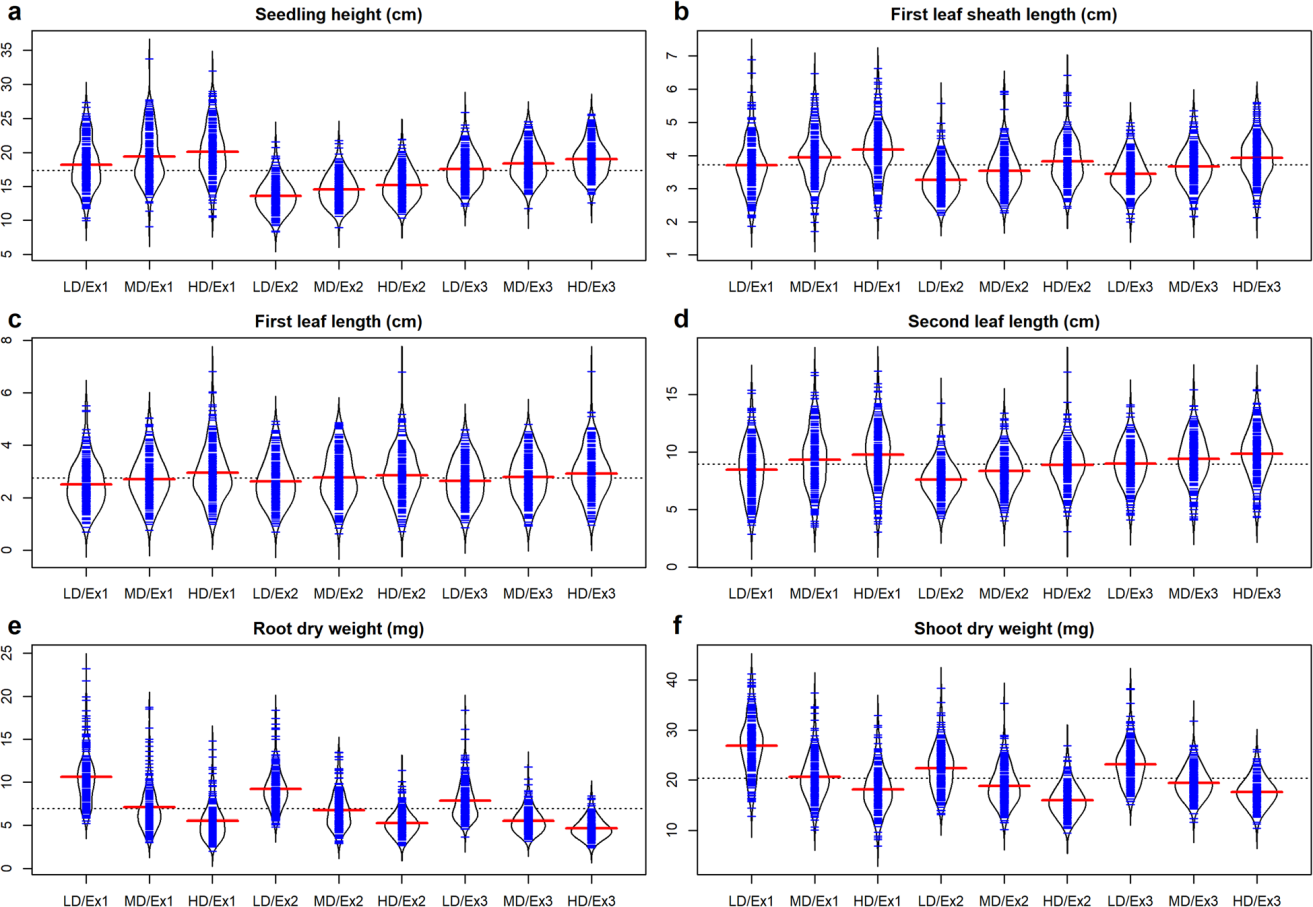
The comparisons of seedling traits performances under three seeding densities. (**a**-**f**) Beanplot and a density plot with symmetrical arrangement of each traits, in which each blue beanline represented each observation value, red line represented the mean value. LD, MD, and HD indicated low, medium, and high seeding density, respectively. Ex1, Ex2 and Ex3 indicated the three experiments, respectively

The difference in seedling traits between the two parental cultivars (‘9311’ and ‘NIP’) was largely affected by seedling density (Additional file 1: Table S2 and Table 1). The two parents differed significantly at *P* ≤ 0.05 for all the six traits under three seeding densities. However, the degree of difference decreased when SLL and RDW were measured as the increasing of seeding density, while the degree of difference between the parents for SH, FLSL, FLL and SDW increased with increasing density. These results indicated the effect of seeding density on seedling traits was not consistent, thus suggesting significant differences of genetic mechanisms for seedling traits between inter-subspecies. In the RIL population, all the seedling traits showed continuous variation and obvious transgressive segregation, following approximate normal distributions. With the increase of seeding density, the average values of RIL population for SH, FLSL, FLL and SLL gradually increased, while the RDW and SDW gradually decreased (Fig. 1). The broad-sense heritabilities ranged from 71.37 to 90.91%, indicating the complexity of the genotypic response to seeding density. The heritability of FLL and SLL were moderate while it was low for RDW and SDW. There was no significant difference in heritability between different densities. The G × E interactions were highly significant (*P* ≤ 0.01) among the three seeding densities, suggesting the effect of seeding density on seedling traits should not be ignored (Table 1). The correlations among different seedling traits showing similar trends between different seeding densities (Additional file 1: Table S3). FLL and SLL showed the highest correlations in both experiments. RDW was negatively correlated with other traits, but it was positively correlated with SDW. Taken together, these results indicated that seedling quality of the parents and RIL population on plastics nursery tray generally declined with the increase of seeding density, and the influences of seeding density on indica and japonica varied on traits. However, little was known about the genetic loci controlling seedling traits that were affected by seeding density, and the distributions of loci that differ in response to seeding density between indica and japonica rice genomes.

**Table 1:** Phenotypic variation of the RIL population of 9311/Nipponbare cross under three seeding densities

Trait^a^	Seeding density^b^	Ex1				Ex2				Ex3				H^e^	G × E^f^
		Range	Mean ± SD^c^	NIP	9311^d^	Range	Mean ± SD^c^	NIP	9311^d^	Range	Mean ± SD^c^	NIP	9311^d^		
SH	LD	9.97—27.37	18.20 ± 3.53	20.16	18.2 **	11.28—24.58	16.59 ± 2.34	18.23	16.63 **	12.11—25.91	17.53 ± 2.47	19.52	16.33 **	79.63	**
	MD	9.07—33.73	19.40 ± 3.91	21.17	18.53 **	11.94—24.73	17.57 ± 2.26	19.07	17.01 **	11.76—24.58	18.38 ± 2.54	21.65	18.23 **	77.76	
	HD	10.48—31.94	20.12 ± 3.85	22.81	19.35 **	13.33—24.96	18.20 ± 2.33	19.68	17.70 **	12.58—25.64	18.99 ± 2.56	22.59	18.59 **	80.81	
FLSL	LD	1.86—6.89	3.71 ± 0.80	3.28	3.77 **	2.20—5.58	3.26 ± 0.55	2.72	3.30 **	2.00—4.99	3.44 ± 0.55	2.89	3.54 **	77.88	**
	MD	1.71—6.47	3.95 ± 0.79	3.58	4.19 **	2.27—5.93	3.54 ± 0.63	3.13	3.60 **	2.15—5.36	3.67 ± 0.58	3.47	4.07 **	82.92	
	HD	2.11—6.63	4.17 ± 0.83	3.74	4.77 **	2.42—6.41	3.83 ± 0.68	3.14	3.83 **	2.13—5.60	3.93 ± 0.65	3.84	4.39 **	80.78	
FLL	LD	0.70—5.51	2.52 ± 0.90	1.42	3.23 **	0.69—4.91	2.62 ± 0.87	1.56	3.15 **	0.68—4.60	2.64 ± 0.81	1.84	2.63 **	88.94	**
	MD	0.76—5.05	2.71 ± 0.87	1.58	3.84 **	0.63—4.86	2.78 ± 0.93	1.57	3.33 **	0.93—4.79	2.79 ± 0.84	1.86	3.08 **	90.91	
	HD	1.00—6.81	2.96 ± 1.05	1.70	3.83 **	0.72—5.17	2.86 ± 0.94	1.67	3.34 **	0.96—5.26	2.93 ± 0.90	1.89	3.67 **	90.15	
SLL	LD	2.85—15.39	8.49 ± 2.36	5.03	11.23 **	4.23—14.25	7.61 ± 1.56	6.14	10.64 **	4.08—14.10	8.99 ± 1.85	8.13	11.00 **	84.36	**
	MD	3.48—16.93	9.33 ± 2.56	6.70	11.55 **	4.02—13.37	8.34 ± 1.71	6.45	10.84 **	4.11—15.43	9.42 ± 2.06	8.56	11.17 **	84.47	
	HD	3.30—17.03	9.77 ± 2.56	7.00	11.81 **	3.07—16.96	8.89 ± 1.87	7.18	11.39 **	4.31—15.41	9.83 ± 2.14	8.61	11.43 **	82.43	
RDW	LD	5.2—23.2	10.6 ± 3.1	9.7	13.8 **	4.8—18.4	9.2 ± 2.3	8.7	12.3 **	3.6—18.4	7.8 ± 2.2	7.6	10.3 **	81.95	**
	MD	3.0—18.7	7.1 ± 2.6	5.7	8.8 **	2.9—13.5	6.7 ± 2.1	6.1	8.1 **	3.2—11.8	5.5 ± 1.4	5.0	7.1 **	71.37	
	HD	2.0—14.8	5.5 ± 2.1	5.3	6.5 **	2.6—11.3	5.3 ± 1.5	5.9	7.0 **	2.4—8.4	4.6 ± 1.1	4.6	6.0 **	78.94	
SDW	LD	12.7—41.2	26.9 ± 5.6	24.9	28.7 **	13.1—38.4	22.4 ± 4.5	19.5	22.7 **	15.1—38.2	23.1 ± 4.1	22.4	24.7 **	77.72	**
	MD	10.1—37.4	20.6 ± 4.4	20.7	24.4 **	10.2—35.3	18.8 ± 3.7	16.3	20.3 **	11.65—31.8	19.4 ± 3.0	19.2	23.5 **	76.63	
	HD	6.9—32.9	18.1 ± 4.4	17.3	21.65 **	9.5—26.9	16.0 ± 3.1	14.8	18.9 **	10.4—26.1	17.6 ± 2.8	16.2	19.8 **	71.46	

^a^Trait. SH, seedling height (cm); FLSL, first leaf sheath length (cm); FLL, first leaf length (cm); SLL, second leaf length (cm); RDW, root dry weight (mg); SDW, shoot dry weight (mg). ^b^ Seeding density, LD, MD, and HD represented low density, medium density, and high density, respectively. ^c^ SD, standard deviation. ^d^ T-test between two parents, * and **, significant at *P* ≤ 0.05 and *P* ≤ 0.01. ^e^ Broad-sense heritability (%). ^f^ G × E, interaction of genotype and seeding density, * and **, significant at *P* ≤ 0.05 and *P* ≤ 0.01

### QTL analysis of seedling traits under three seeding densities

QTL analysis based on SNP bin map under three seeding densities in three experiments identified 37 QTLs. Four QTLs for SH, 9 QTLs for FLSL, 6 QTLs for FLL, 5 QTLs for SLL, 5 QTLs for RDW and 8 QTLs for SDW were identified above the LOD thresholds under three seeding densities (Additional file 1: Table S4). We further analyzed in detail the QTLs that detected in at least two experiments (Table 2).

**Table 2:** Comparative analysis of QTLs for seedling traits under three seeding densities

Chr	Position ^a^	QTL	Traits/ experiment^b^	LD			MD			HD		
				Add^c^	LOD	Var^d^	Add^c^	LOD	Var^d^	Add^c^	LOD	Var^d^
1	bin72	*qRDW*_*1.1*_	RDW/Ex1	−1.02	4.73	8.25						
		*qRDW*_*1.1*_	RDW/Ex2	−0.61	3.79	6.82	−0.48	3.45	5.08			
		*qRDW*_*1.1*_	RDW/Ex3	−0.63	7.42	7.64	−0.4	4.79	8.09			
	bin191	*qRDW*_*1.2*_	RDW/Ex1							−0.47	3.28	4.7
		*qRDW*_*1.2*_	RDW/Ex2							− 0.35	4.45	5.72
	bin311	*qSH*_*1.1*_	SH/Ex1	0.68	6.42	7.12	1.09	9.45	7.34	1.10	8.69	7.35
		*qSH*_*1.1*_	SH/Ex2	0.99	6.74	16.0	1.07	13.52	20.2	1.07	13.46	19.07
		*qSH*_*1.1*_	SH/Ex3	0.96	14.70	13.46	1.13	17.5	17.91	1.12	15.53	17.24
		*qFLSL*_*1.1*_	FLSL/Ex1	0.16	4.79	6.75	0.11	3.82	4.12	0.13	4.13	4.75
		*qFLSL*_*1.1*_	FLSL/Ex2	0.18	5.22	9.74	0.23	8.53	11.73	0.25	8.91	11.99
		*qFLSL*_*1.1*_	FLSL/Ex3	0.18	6.27	10.68	0.19	10.31	9.85	0.2	8.23	8.52
2	bin411	*qFLL*_*2.1*_	FLL/Ex1				−0.31	4.87	12.97	−0.35	3.84	10.86
		*qFLL*_*2.1*_	FLL/Ex2				− 0.30	4.11	10.6			
		*qFLL*_*2.1*_	FLL/Ex3				− 0.31	5.84	13.41	−0.35	4.67	14
		*qSLL*_*2.1*_	SLL/Ex1				−0.55	3.28	9.55	−0.56	3.98	9.73
		*qSLL*_*2.1*_	SLL/Ex3	−0.50	3.36	7.47	−0.58	3.4	8.01	−0.61	3.83	8.16
		*qSDW*_*2.1*_	SDW/Ex2	1.13	4.58	6.29	0.90	3.32	5.83	0.88	3.42	7.77
		*qSDW*_*2.1*_	SDW/Ex3	0.73	3.66	5.11						
	bin560	*qFLL*_*2.2*_	FLL/Ex2	−0.29	3.13	11.48				−0.29	3.62	8.69
		*qFLL*_*2.2*_	FLL/Ex3	−0.28	6.54	11.82				−0.30	3.25	10.54
3	bin650	*RDW*_*3.1*_	RDW/Ex1	−0.46	3.44	5.07						
		*RDW*_*3.1*_	RDW/Ex2	−0.67	4.16	8.3	−0.57	3.87	7.03			
		*RDW*_*3.1*_	RDW/Ex3	−0.46	4.11	4.3	−0.25	3.75	3.53			
	bin758	*qSLL3.1*	SLL/Ex2							−0.59	4.51	8.16
		*qSLL3.1*	SLL/Ex3				−0.77	3.3	11.8	−0.85	3.4	12.28
4	bin1135	*qSH*_*4.1*_	SH/Ex1	−0.92	3.66	6.73				−1.07	4.51	7.81
		*qSH*_*4.1*_	SH/Ex3	−0.52	4.14	4.41	−0.56	4.61	4.83	−0.52	3.52	4.13
		*qSDW*_*4.1*_	SDW/Ex1	−2.07	6.08	9.49	−1.36	3.96	8.81	−1.27	4.07	8.3
		*qSDW*_*4.1*_	SDW/Ex2	−0.97	5.36	4.61	−0.73	4.39	3.79	−0.58	5.07	3.49
		*qSDW*_*4.1*_	SDW/Ex3	−1.21	6.06	8.79	−0.96	6.39	10.14	−0.77	3.81	7.6
5	bin1218	*qSDW*_*5.1*_	SDW/Ex2	0.91	4.28	4.08						
		*qSDW*_*5.1*_	SDW/Ex3	0.74	4.89	5.2						
	bin1281	*qFLSL*_*5.1*_	FLSL/Ex1							−0.20	5.93	5.82
		*qFLSL*_*5.1*_	FLSL/Ex2				−0.10	3.56	4.44	−0.09	4.00	4.54
		*qFLSL*_*5.1*_	FLSL/Ex3							−0.11	3.92	4.84
	bin1325	*qSDW*_*5.2*_	SDW/Ex1				−1.55	3.92	11.63			
		*qSDW*_*5.2*_	SDW/Ex3	−0.99	3.81	5.91	−0.88	5.75	8.67			
6	bin1565	*qFLSL*_*6.1*_	FLSL/Ex1	−0.25	3.25	9.49						
		*qFLSL*_*6.1*_	FLSL/Ex3				−0.13	4.44	4.46			
		*qFLL*_*6.1*_	FLL/ Ex1	−0.38	9.18	17.98	−0.38	9.58	18.67	−0.41	7.57	15.08
		*qFLL*_*6.1*_	FLL/Ex2	−0.36	8.22	17.12	−0.42	7.48	20.12	−0.40	6.47	17.2
		*qFLL*_*6.1*_	FLL/Ex3	−0.36	7.05	19.83	−0.41	9.18	23.74	−0.42	10.16	20.32
		*qSLL*_*6.1*_	SLL/Ex1	−0.75	10.01	19.18	−0.74	6.05	15.88	−0.72	7.72	15.08
		*qSLL*_*6.1*_	SLL/Ex2	−0.52	6.96	11.13	−0.59	7.57	11.76	−0.45	7.13	5.84
		*qSLL*_*6.1*_	SLL/Ex3	−0.71	12.2	14.64	−0.80	6.57	15.13	−0.82	7.17	14.01
9	bin2139	*qFLL*_*9.1*_	FLL/Ex2	−0.28	5.06	10.73	−0.36	6.65	14.92	−0.34	3.99	11.87
		*qFLL*_*9.1*_	FLL/Ex3	−0.32	4.33	15.33				−0.35	5.67	13.85
	bin2176	*qFLL*_*9.1*_	FLL/Ex1	−0.34	7.41	14.45	−0.31	6.66	13.03	−0.33	4.91	10.07
		*qFLL*_*9.1*_	FLL/Ex3	−0.29	4.62	13.14	−0.32	5.77	14.81	−0.32	4.56	11.87
11	bin2518	*qSH*_*11.1*_	SH/Ex1	−1.13	5.74	10.13	−1.13	4.92	8.39	−0.95	4.37	6.58
		*qSH*_*11.1*_	SH/Ex3	−0.51	4.3	4.19	−0.51	3.62	3.99	−0.54	4.36	4.51
		*qSDW*_*11.1*_	SDW/Ex1	−2.06	3.79	9.38	−1.53	5.05	11.24	−1.30	4.4	8.78
		*qSDW*_*11.1*_	SDW/Ex3	−1.05	5.18	6.72	−0.90	4.82	9.04	−0.75	3.38	7.36
12	bin2760	*qFLSL*_*12.1*_	FLSL/Ex1				0.12	4.12	4.63			
		*qFLSL*_*12.1*_	FLSL/Ex2	0.10	4.02	4.28						
		*qFLSL*_*12.1*_	FLSL/Ex3				0.12	6.46	4.78			

^a^The position of the LOD peak of each QTL. ^b^ Trait/experiment. SH, seedling height; FLSL, first leaf sheath length; FLL, first leaf length; SLL, second leaf length; SDW, shoot dry weight; RDW, root dry weight. Ex1, Ex2 and Ex3 indicated the three experiments, respectively. ^c^ Addictive effect. Positive values indicate that the allele from Nip increase trait values. ^d^ Variance (%) explained by the QTL

For seedling height, three QTLs distributed on chromosomes 1 (*qSH*_*1.1*_), chromosomes 4 (*qSH*_*4.1*_) and chromosomes 11 (*qSH*_*11.1*_) were both identified in at least two seeding densities, respectively. At *qSH*_*1.1*_, the allele from ‘NIP’ increased the seedling height, while the allele from ‘9311’ had positive effect at *qSH*_*4.1*_ and *qSH*_*11.1*_. Moreover, the phenotypic variances explained by *qSH*_*1.1*_ slightly increased with the increase of seedling density. For FLSL, four QTLs were detected at chromosomes 1, 5, 6 and 12 (*qFLSL*_*1.1*_, *qFLSL*_*5.1*_, *qFLSL*_*6.1*_ and *qFLSL*_*12.1*_), respectively. *qFLSL*_*1.1*_ was detected in both three seeding densities, while *qFLSL*_*5.1*_ was specially detected at MD and HD. On the other hand, *qFLSL*_*6.1*_ and *qFLSL*_*12.1*_ were only identified at LD and MD. For *qFLSL*_*5.1*_, the positive genotypes came from ‘9311’, while for the other two QTL (*qFLSL*_*1.1*_ and *qSDW*_*12.1*_), the positive genotypes came from ‘NIP’. For first leaf length, all the five QTLs (*qFLL*_*2.1*_, *qFLL*_*2.2*_, *qFLL*_*6.1*_, *qFLL*_*9.1*_ and *qFLL*_*9.2*_) were identified in at least two seeding densities, in which *qFLL*_*6.1*_ explained maximum phenotypic variances of 17.98, 18.67 and 15.08% at LD, MD and HD in Ex1, respectively. Furthermore, the alleles from ‘9311’ for all QTLs increased first leaf length. The QTLs for second leaf length were detected on chromosome 2 (*qSLL*_*2.1*_), chromosome 3 (*qSLL*_*3.1*_) and chromosome 6 (*qSLL*_*6.1*_). *qSLL*_*6.1*_ explained the largest phenotypic variation and existed at all three seeding densities, while *qSLL*_*3.1*_ was only detected at MD and HD. All the three QTLs of SLL functioned in the same direction, with the allele from ‘9311’ increasing the phenotypic value. Three QTLs were identified for RDW on chromosomes 1 (*qRDW*_*1.1*_, *qRDW*_*1.2*_) and chromosomes 3 (*qRDW*_*3.1*_). Interestingly, *qRDW*_*1.1*_ and *qRDW*_*3.1*_ were detected in LD and MD, while *qRDW*_*1.2*_ was detected only in HD. Besides, the allele of ‘9311’ increased RDW for all three QTLs. Five QTLs for shoot dry weight (*qSDW*_*2.1*_, *qSDW*_*4.1*_, *qSDW*_*5.1*_, *qSDW*_*5.2*_ and *qSDW*_*11.1*_) were identified on chromosomes 2, 4, 5(2), and 11. For *qSDW*_*2.1*_, the positive genotype came from ‘NIP’, while for the other four QTL (*qSDW*_*4.1*_, *qSDW*_*5.1*_, *qSDW*_*5.2*_ and *qSDW*_*11.1*_), the positive genotype came from ‘9311’. Two QTLs (*qSDW*_*4.1*_ and *qSDW*_*11.1*_) were detected at both three seeding densities, while *qSDW*_*5.1*_ and *qSDW*_*5.2*_ were detected only at LD and MD.

**Fig. 2:**
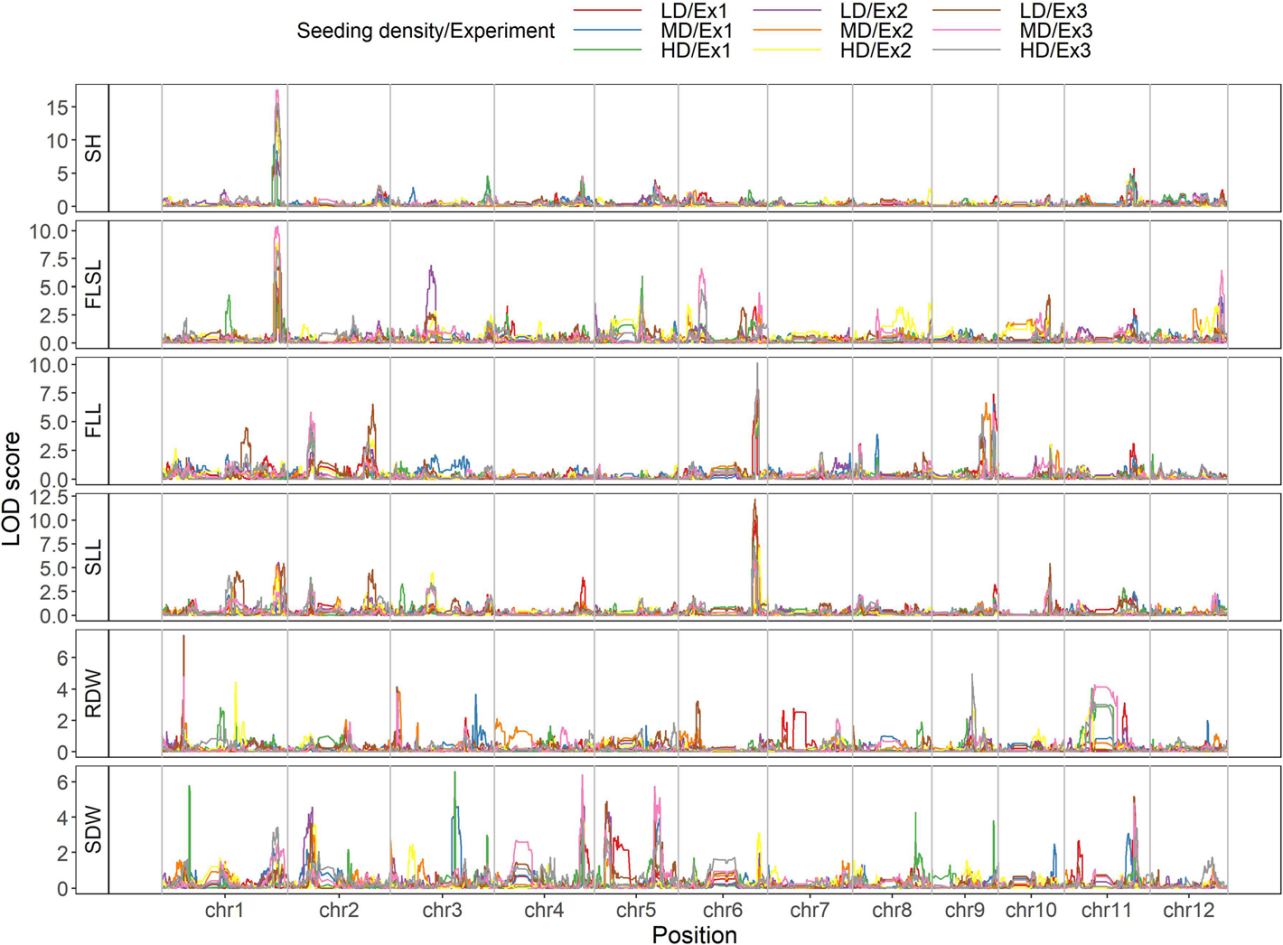
QTL curves and peaks for seedling traits under three seedling densities. LD, low density; MD, medium density; HD, high density; Ex1, Ex2 and Ex3 indicated the three experiments, respectively. SH, seedling height; FLSL, first leaf sheath length; FLL, first leaf length; SLL, second leaf length; RDW, root dry weight; SDW, shoot dry weight

QTL hotspot is a region where multiple traits were co-located. There were five QTL hotspots (bin311, bin411, bin1135, bin1565 and bin2518) identified clustered within genomic regions on chromosomes 1, 2, 4, 6 and 11 (Table 2 and Fig. 2). However, it was not clear whether these five QTL hotspots were single locus with pleiotropic effects on the multiple traits, or a group of tightly linked loci. Further analyses revealed some noteworthy features about these regions. Three of these hotspots, bin311, bin1135 and bin2518 showed almost consistent effects across the three seeding densities, while bin411 mainly detected at MD and HD for FLL and SLL, with minor effects at LD. However, bin411 functioned at LD for SDW. The QTL hotspot at bin1565 on chromosome 6 were simultaneously detected for FLL and SLL at all three seeding densities, however, this hotspot was only detected for FLSL at LD and MD. These results suggest that bin311, bin1135 and bin2518 were not sensitive to seeding density, while bin411 and bin1565 appeared to be QTL-specific for seedling density. Another interesting finding was that the effects of QTL hotspots for the multiple seedling traits showed diversity. For bin311, the allele from ‘NIP’ increased trait values, with the additive effects in the same direction for SH and FLSL. For the other three QTL hotspots (bin1135, bin1565 and bin2518), the allele from ‘9311’ increased trait values, and consistent among those multiple traits. However, the additive effect of bin411 showed the opposite direction, with positive effects for SDW and negative effects for FLL and SLL. All the five QTL hotspots produced considerable individual effects on the seedling traits at the three seedling densities. These results were consistent with the observation that all the traits displayed continuous variation in the RIL population, implying that seedling traits in rice were contributed by many loci with small effects. Furthermore, the existence of specific QTLs at different seeding densities also revealed the differences in seedling traits of indica rice and japonica rice in response to seeding densities.

### Genome-wide additive and epistatic effects

**Fig. 3:**
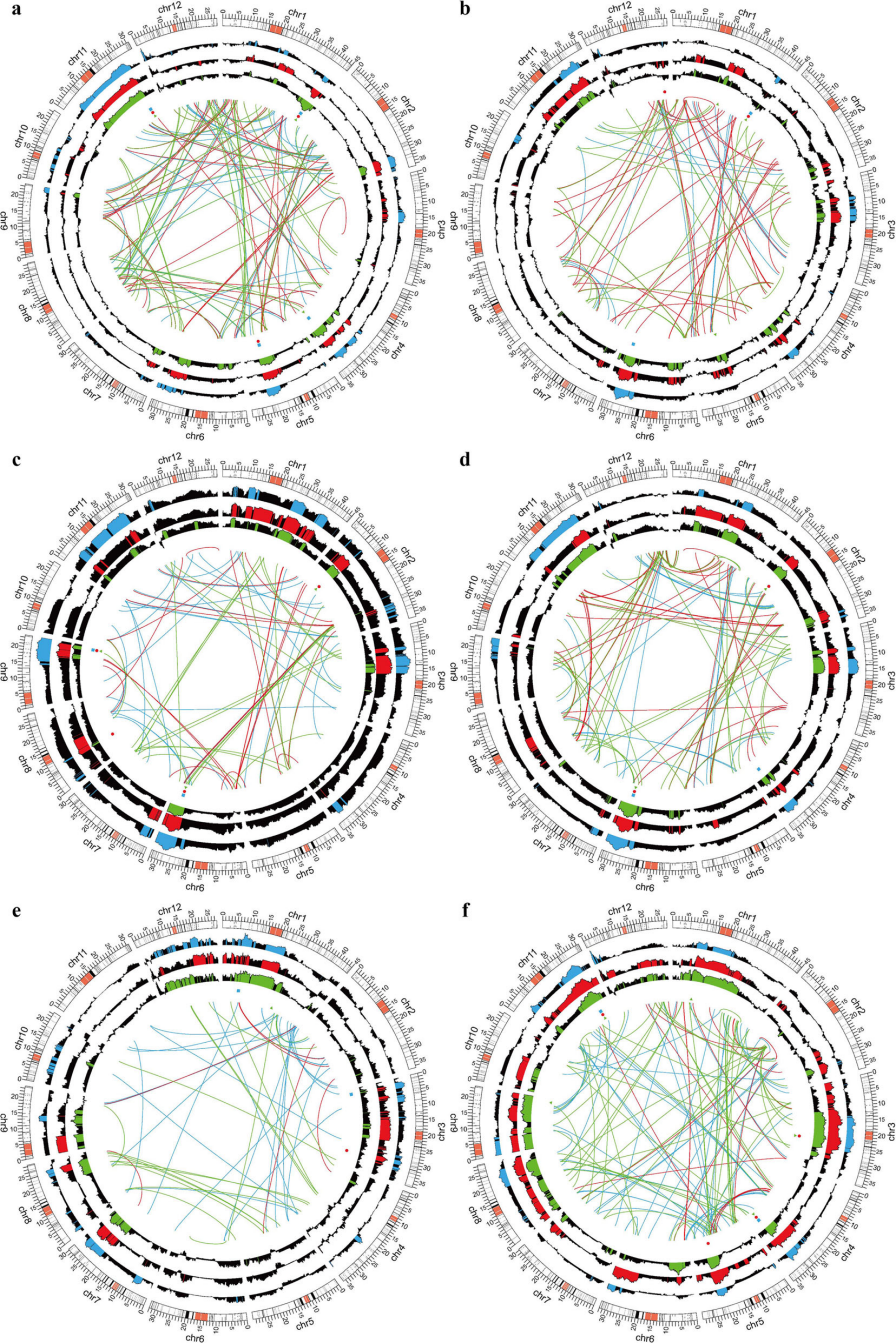
The comparisons of genome-wide distributions of QTLs, additive and epistasis effects for seedling traits under three seeding densities in Ex1. **a** seedling height; **b** first leaf sheath length; **c** first leaf length; **d** second leaf length; **e** root dry weight; **f** shoot dry weight. The outermost circle represented the distribution of bin markers on 12 chromosomes. The histogram plots from the second to forth circle were the distributions of additive effects under LD, MD and HD, respectively. The blue, red and green bars represented the bins with significant additive effects under LD, MD and HD, respectively, and the black bars indicated non-significant additive effects. The blue squares, red circle and green triangle indicated the locations of QTLs detected under LD, MD and HD, respectively. The blue, red and green lines in the cycle indicated significant interactions under LD, MD and HD, respectively

Although the main-effect QTLs of young seedlings traits related to machine transplanting under different seeding densities had been fully explored. We did not have a complete understanding of the contributions of the ‘9311’ and ‘Nip’ genomes to these traits at whole-genome level. Genome-wide additive and epistatic effects for each trait at three seeding densities were estimated based on the high-density bin map. The genome-wide additive and epistatic effects and the distribution of QTLs in Ex1 were detailed showed in Fig. 3.

**Fig. 4:**
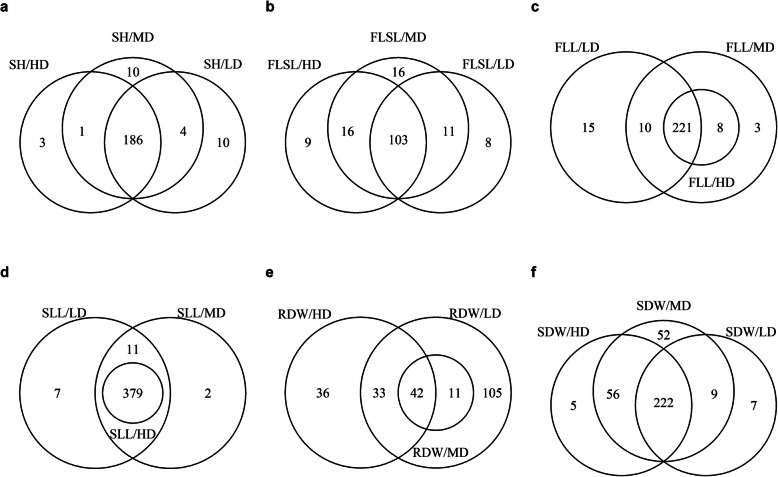
Venn diagram of number of significant bins detected for six seedling traits under three seeding densities. **a** SH, seedling height; **b** FLSL, first leaf sheath length; **c** FLL, first leaf length; **d** SLL, second leaf length; **e** RDW, root dry weight; **f** SDW, shoot dry weight. LD, low density; MD, medium density; HD, high density

Single-locus positive and negative additive effects were extensively distribution among all the seedling traits, however, the contributions of the allele from ‘9311’ and ‘NIP’ differed across the traits and seeding densities (Additional file 1: Table S5). One of the most noticeable results was that the number of negative additive effect bins was more than that of positive additive effect bins for most traits, and the trend was consistent under different seeding densities (Table 3). However, there were a few exceptions, the number of positive and negative additive effect bins was approximately equal for SH and FLSL in Ex2 and Ex3. Besides, there were no bins with significant positive effect for some traits at both three seeding densities, such as FLL in all three experiments, SLL in Ex1, and RDW in Ex2 (Table 3). These results implied that the allele from ‘9311’ contributing to increase seedling traits performance were widely genomic distribution. The comparisons of the distribution of additive effects under three seeding densities showed that the number of bins simultaneously detected under the three seeding densities took the advantage for all the other traits, except for RDW, the number of bins detected at the LD condition dominated. Another noteworthy result was that the bins that detected under HD were all contained under MD for FLL, while the bins detected under MD were contained under LD for RDW, and the bins detected under HD were included under both LD and MD for SLL (Fig. 4).

**Table 3:** Summary of significant additive effects and two-locus interactions under three seeding densities

Trait^a^	Seeding density^b^	Ex1				Ex2				Ex3			
		PAE^c^	NAE^d^	PEE^e^	NEE^f^	PAE	NAE	PEE	NEE	PAE	NAE	PEE	NEE
SH	LD	36	488	12	42	80	99	0	33	166	363	4	50
	MD	62	529	13	35	82	86	0	41	137	228	2	64
	HD	82	465	6	42	73	109	1	53	166	159	3	79
FLSL	LD	104	225	5	12	168	294	1	79	173	183	1	88
	MD	85	435	9	35	271	245	0	100	161	257	3	91
	HD	74	382	12	19	351	243	0	118	138	267	2	120
FLL	LD	0	323	22	9	0	335	8	36	0	466	15	25
	MD	0	497	16	9	0	278	25	17	0	553	18	31
	HD	0	535	11	13	0	202	3	53	0	379	6	34
SLL	LD	0	437	4	29	39	321	1	40	21	215	4	62
	MD	0	547	18	27	48	391	0	68	26	252	8	103
	HD	0	491	10	31	19	461	0	68	23	381	9	99
RDW	LD	1	425	1	26	0	650	1	5	6	125	5	11
	MD	0	345	2	4	0	184	0	3	0	99	2	9
	HD	0	471	0	18	0	300	0	12	17	83	0	2
SDW	LD	24	484	8	41	63	334	6	15	74	282	8	29
	MD	1	525	5	18	42	108	1	12	19	559	5	14
	HD	5	416	2	55	21	97	4	14	35	440	7	18

^a^Trait, SH, seedling height; FLSL, first leaf sheath length; FLL, first leaf length; SLL, second leaf length; RDW, root dry weight (mg); SDW, shoot dry weight (mg). ^b^ Seeding density, LD, MD, and HD represented low density, medium density, and high density, respectively. ^c, d^ PAE and NAE, indicated the number of bins with positive additive effect and negative additive effect, respectively. Positive values indicate that alleles from Nip were in the direction of increasing the trait scores, and negative values indicate that alleles from 9311 are in the direction of increasing the score. ^e, f^ PEE, NEE indicated the number of two-locus interactions with significant positive and negative epistatic effects, respectively

Digenetic interactions of each bin pairs across the entire genome at 0.0001 probability level were shown in Additional file 1: Table S6. Less numbers of digenic interactions contributing for seedling traits, explaining lower proportions of phenotypic variance than single-locus analysis (Table 3). However, most of the interaction pairs individually accounted for more than 6% of the genotypic variation, which were not less than some main-effect QTLs. Significant interactions involve many marker loci, most of which were not detected in the single-locus analysis. However, there were still some QTLs found to interact with two or more other locus. The *qFLSL*_*12.1*_ was identified simultaneously interacted with bin1368, bin1845 and bin2499 at HD. The *qFLL*_*6.1*_ was detected to interacted with *qFLL*_*2.2*_ and *qFLL*_*9.1*_ at HD, and they were both showed positive epistasis effects. However, the interactions might change under different seeding densities. For example, the *qFLL*_*6.1*_ was detected to interacted with bin1938 at LD in Ex1. Similarly, *qSLL*_*2.1*_ at QTL hotspot bin411 interacted with bin2640 at LD, while it interacted with bin1290 at HD in Ex1. QTL for SDW at bin90 interacted with bin892 at LD and MD, while it interacted with bin431 and bin1302 at HD in Ex1 (Fig. 3). Another result worth noting was that the number of interactions with negative epistasis effects were predominant for SH, FLSL, SLL, RDW and SDW at three seeding densities, except for FLL (Table 3 and Additional file 1: Table S6). Taken together, genome-wide additive and two-locus epistasis suggested a dynamic of the genetic control underlying different seeding densities.

## Discussion

Our conclusions implicated the complex of genetic basis of rice seeding traits under different seeding densities which was only beginning to be acknowledged. In considering the results previous researches, we realized that the fundamental genetic mechanisms of seedling trait in rice were so complex, and partially caused by the diversity of rice germplasm [37, 38, 43]. Therefore, we compared identified QTL regions with previously reported QTL loci in other mapping populations and germplasm materials. *qRDW*_*1.1*_ were located at bin72, which was previously reported affecting root number and root dry weight and length of mesocotyl [46–48]. The QTL hotspot at bin311 (*qSH*_*1.1*_, *qFLSL*_*1.1*_) were overlapped with QTLs related to shoot length in previous reports and a candidate gene *Os01g0904700* was identified responsible for increasing shoot length [35, 37, 49]. However, another main-effect QTL on chromosome 3 reported to increase seedling height and leaf sheath length were identified as *OsGA20ox1*, which was not detected in our research [41, 49]. The QTL *qSDW*_*5.1*_ were overlapping with the corresponding seedling vigor QTL mapped to the same locations [50, 51]. Besides, another QTL hotspots on chromosome 6 (*qFLSL*_*6.1*_, *qFLL*_*6.1*_ and *qSLL*_*6.1*_) and *qFLL*_*9.1*_ coincided with those previously reported QTLs related to shoot dry weight and shoot length in both directions of additive effects and the locations [37]. The co-location of QTLs for rice seedling traits indicated the reliability of these QTLs with common effects and could be found in different studies even in different populations or germplasm resources.

One of the highlights of our research was conducting the seeding density experiments directly on the rice seedling tray, which was closely related to rice production. Although the quality of rice trays and substrates were strictly controlled in our research, the fluctuations of light, temperature and other climatic factors between years might still impact seedling growth. Nevertheless, some intriguing findings had been revealed. The growth of rice seedlings was significantly influenced by seeding density, as indicated in phenotypic data (Table 1). It showed that the seedling traits of the two parents exhibited similar trends as the seeding density increased, including SH, FLSL and SLL increases, SDW and RDW decreases. However, the differences of RDW gradually decreased, while SH, FLSL and SDW gradually increased as the seeding density increased, indicating the difference between aboveground and underground parts response to seeding density between ‘9311’ and ‘NIP’. The average traits performances of RIL population showed similar trends of the parents as the seeding density increased, and normally distributed on the seedling tray. Moreover, the interactions between seeding densities and genotypes had a greater influence on seedling traits indicating that the gene action related to seedling traits should be evaluated under certain densities. QTL analysis results indicated that ‘9311’ contributed more QTL loci enhancing trait performances than ‘NIP’ for all six traits. The results of whole-genome additive effects under three seeding densities also supported that more alleles responding to seeding density can be found in indica subspecies than in japonica subspecies. Although there were considerable overlaps in the genome-wide distribution of significant additive effects for the traits under the three seeding densities, such as SH, FLSL, FLL and SLL, special loci at certain seeding density still existed, which suggested that these loci were sensitive to seeding density and worthy further investigation (Fig. 4). The prevalence of negative epistasis effects indicated that the complementary two-locus homozygotes may not have marginal advantages over the means of the two parental genotypes. Moreover, some QTLs were found to interact with two or more loci, which suggested the main effects of QTLs were likely to be embedded in the interactions the rest of whole genome. Some interactions were specifically detected at certain seeding density, suggesting epistasis effect were also sensitive to seeding density. Thus, it may not be appropriate to interpret the single-locus marginal effects without specifying the genotypes and seeding density of the counterpart.

Another highlight was that several QTLs were specifically detected at certain seeding density (Table 2). Among 37 QTLs for six traits detected under the three seeding densities in our study, only 12 QTLs were detected in both three seeding densities. In other words, the rest QTLs were seedling density specific. This strongly suggested that QTL detection for seedling traits depended on the specific seeding density. For SH, all the three QTLs (*qSH*_*1.1*_, *qSH*_*4.1*_ and *qSDW*_*11.1*_) showed consistent effects on the traits across all three seeding densities, implying that the effect of seeding density on seedling height had universal effects. However, for FLSL, only one QTL (*qFLSL*_*1.1*_) was detected across the three seeding densities. *qFLSL*_*5.1*_ was specifically detected at bin1281 region under MD and HD, while *qFLSL*_*6.1*_ and *qFLSL*_*12.1*_ specifically detected at LD and MD. Besides, one QTL hotspot for FLL and SLL at bin411 were detected at MD and HD, except for LD, and the allele of ‘9311’ increased the trait values. Two QTLs for RDW, *qRDW*_*1.1*_ and *qRDW*_*3.1*_ were both detected under LD and MD, rather than HD. Among all the QTL identified for SDW (*qSDW*_*2.1*_, *qSDW*_*4.1*_, *qSDW*_*5.1*_, *qSDW*_*5.2*_ and *qSDW*_*11.1*_), *qSDW*_*2.1*_, *qSDW*_*4.1*_ and *qSDW*_*11.1*_ showed consistent effects on the traits across all three seeding densities, while *qSDW*_*5.1*_ and *qSDW*_*5.2*_ only detected at LD and MD, respectively. However, none of QTLs for RDW overlapped among the three seeding densities. Root dry weight were more easily affected by seeding density compared with shoot related traits, suggesting that there might be different mechanisms involved in root and shoot development under certain seeding density. However, the correspondences between those specifically detected QTLs and responses to the seeding density could not be clarified. Although the physiological and biochemical mechanisms of these QTLs have not been elucidated, specific QTLs might be useful for rice seedling improvement during mechanical transplanting.

**Fig. 5:**
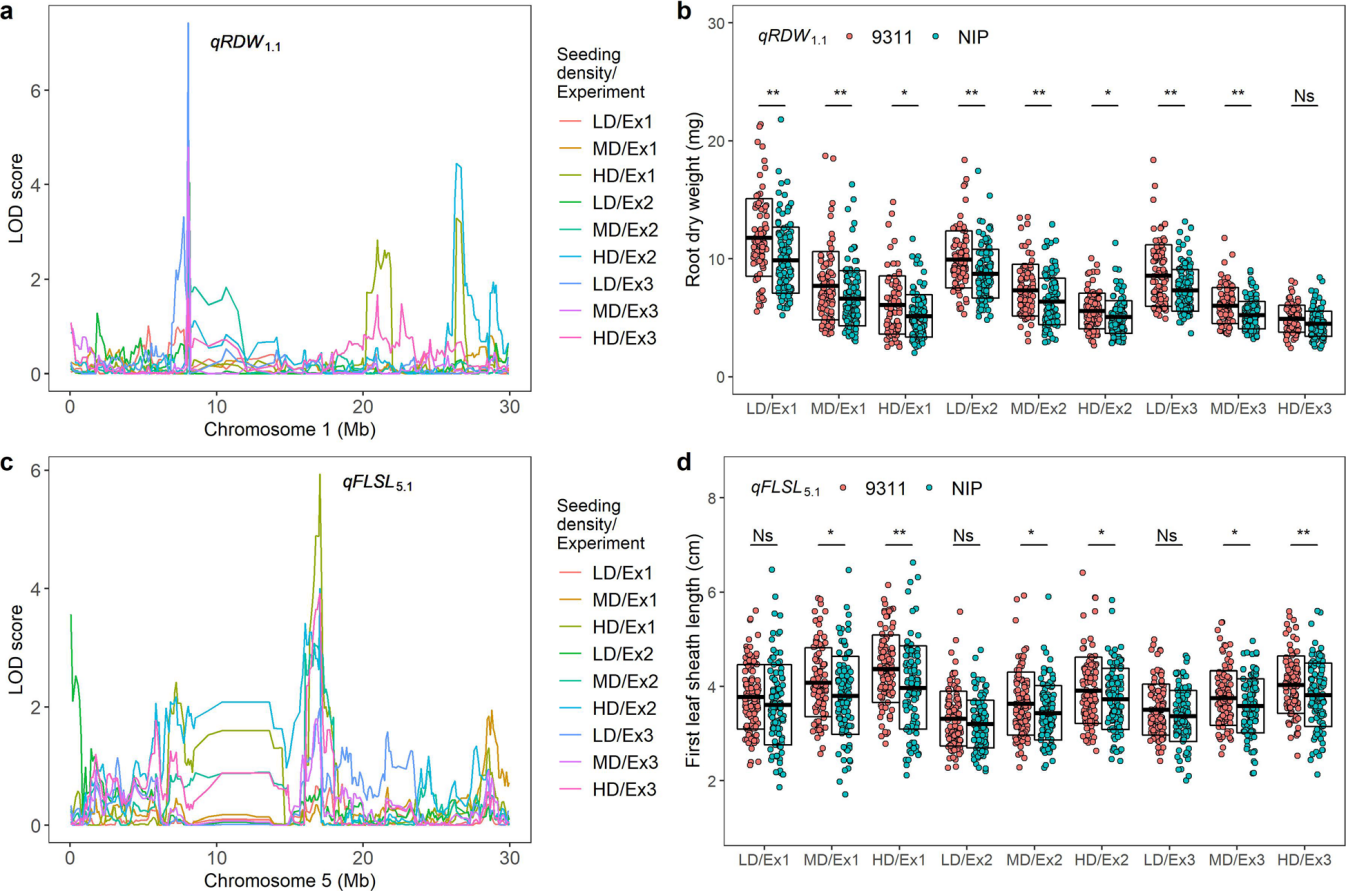
QTLs specifically detected at certain seeding density. **a** lod curves and peak of *qRDW*_*1.1*_; **b** Comparisons of root dry weight between 9311 and Nip genotypes under three seeding densities. **c** lod curves and peak at three seeding densities of *qFLSL*_*5.1*_; **d** Comparisons of first leaf sheath length between 9311 and Nip genotypes at three densities. Ns, * and ** indicated no significant difference, significant at *P* ≤ 0.05 and *P* ≤ 0.01, respectively

Rice breeding strategies had been focused on yield, insects and diseases resistance, high nutrient efficiency and drought resistance in recent decades [52], less attention had been paid to improve the efficiency of mechanical transplanting through seedling traits improvements. The selection strategies of optimal seeding density for hybrid rice and conventional rice varieties were different from each other during mechanical transplanting [5]. Under high seeding density, indica rice grew faster than japonica rice seedlings, the decrease of seedling quality leads to increase of high-order tillering and yields decline [53], which supported our results that there were more QTLs and additive loci sensitive to seeding density in ‘9311’ as compared with ‘NIP’. Due to the higher seed cost of hybrid rice and seedling heterosis [54], the advantages of hybrids can only be exerted by low-density seeding, so the seedling quality should be improved as much as possible under the low-density seeding condition to ensure the completion of machine transplanting [17, 26]. Conventional indica rice variety required high-density seeding because of its lower seed cost and poor seedling quality than hybrid rice [5]. However, high density lead to further decrease of individual quality, the alleles and QTLs that exerted their functions under high-density seeding conditions were important for conventional indica rice seedling quality improvement. In the present study, QTLs specifically detected at low or high seeding densities may be important potential targets for improving seedling traits to adapt to machine transplanting. The interweaving force of rice seedlings was an important limiting factor for hybrid rice mechanical transplanting, which required enough root system to form firm structures on the premise of low seeding density [7]. Therefore, alleles and QTLs that functioned on root related traits under low seeding density were the best candidates to improve the seedling quality of hybrid rice seedlings. The *qRDW*_*1.1*_ would be an ideal target for hybrid rice seedling improvement. The allele from ‘9311’ strongly increased the root dry weight, as the seeding density decreased (Fig. 5a and b). Another potential target was *qFLSL*_*5.1*_, which was specifically detected at high seeding density, and the allele of ‘9311’ increased the first leaf sheath length (Fig. 5c and d). Although both the allele of ‘NIP’ and ‘9311’ increased first leaf sheath length as seeding density increased, the ‘9311’ allele was more sensitive to seeding density than the ‘NIP’ allele, resulting in a significant increase in FLSL of ‘9311’ with the increase of seeding density. Thus, the allele of ‘NIP’ could improve first leaf sheath length for conventional indica rice varieties under high-density seeding conditions.

Those QTLs detected under specific seeding density conditions may be involved in the shade avoidance response of rice seedlings. Detailed analysis of the regions of these QTLs revealed several candidate genes, involving in phytohormone signals, including abscisic acid (ABA), gibberellin, auxin, and ethylene and some abiotic-stresses related genes (Table 4). *qRDW*_*1.1*_ were identified on chromosome 1. This region was found to contain five putative genes, two of them were involved in auxin (*LOC_Os01g13520*) [55, 56] and ABA (*LOC_Os01g13530*) [57] signals. *NLP3* (*LOC_Os01g13540*) played a major role in nitrate uptake, translocation and signaling in rice root [58]. Both *LOC_Os01g13570* [59] and *LOC_Os01g13740* [60] participated in abiotic stress response. Similarly, in the *qFLL*_*2.1*_ region, three candidate genes including *OsbZIP19* (*LOC_Os02g14910*), *OsCNGC1* (*LOC_Os02g15580*) and ABA receptor *PYL3* (*LOC_Os02g15640*) were identified. *OsbZIP19* was upregulated in overexpression of *ONAC022* plants and played a role against abiotic stresses in rice [61]. *OsCNGC1* [62] and *PYL3* [63] were both triggered by ABA signals. Four genes were found within the *qFLSL*_*5.1*_ region. *OsABI4* (*LOC_Os05g28350*) was the link between ABA and cytokinin signal transduction [64], likely to be the best candidate in this region. *LOC_Os05g28500*, a pentatricopeptide-repeat protein (PPR), specifically expressed in rice seedling and 20-day-old leaves, induced under the biotic and abiotic stresses [65]. *LOC_Os05g28730*, encoding a C3HC4-type zinc finger domain containing protein [66] and *LOC_Os05g28740* (universal stress protein) [67] were both reported to involved in abiotic stress response. Detailed functional analysis of these genomic regions and candidate genes through construction and evaluation of near-isogenic lines would further improve the understanding of genetic basis of seedling traits related to seeding densities as well as putting into practice according to variety types and cropping systems during mechanical transplanting.

**Table 4:** List of candidate genes for QTLs identified in specific seeding density

QTLs	Chromosome	MSU locus ID	Gene annotation	Reference
*qRDW*_*1.1*_	1	*LOC_Os01g13520*	*OsARF16*, auxin response factor 1-like	[55, 56]
	1	*LOC_Os01g13530*	*ABIL3*, Abl interactor-like protein 3, expressed	[57]
	1	*LOC_Os01g13540*	*NLP3*, Nodule inception protein-like protein 3	[58]
	1	*LOC_Os01g13570*	Coding for phosphoglycerate mutase, expressed	[59]
	1	*LOC_Os01g13740*	*OsGLK2*, probable transcription factor *GLK2*	[60]
*qFLL*_*2.1*_	2	*LOC_Os02g14910*	*OsbZIP19*, putative bZIP transcription factor RF2b	[61]
	2	*LOC_Os02g15580*	*OsCNGC1*, Cyclic nucleotide-gated channels family, Group I	[62]
	2	*LOC_Os02g15640*	Abscisic acid receptor *PYL3*, PYR1-like protein 3	[63]
*qFLSL*_*5.1*_	5	*LOC_Os05g28350*	*OsABI4*, Ethylene-responsive transcription factor *ABI4*	[64]
	5	*LOC_Os05g28500*	Pentatricopeptide-repeat proteins (PPRs)	[65]
	5	*LOC_Os05g28730*	Zinc finger, C3HC4 type domain containing protein	[66]
	5	*LOC_Os05g28740*	Universal stress protein domain-containing protein, putative	[67]

## Conclusions

In this study, we employed the technique of bowl-shaped blanket-like seedling to assess seedling traits variations of a RIL population derived from ‘9311’ and ‘Nipponbare’ under three seeding densities. Our results revealed the differences between indica rice and japonica rice seedling traits in response to seeding density. Several genomic regions containing QTL hotspots involved in different traits and specific QTLs (such as *qRDW*_*1.1*_ and *qFLSL*_*5.1*_) in diverse seeding densities had been detected. Genome-wide additive and two-locus epistasis suggested a dynamic of the genetic control underlying different seeding densities. Novel QTLs functioned under specific seeding density could be potential targets for marker-assisted selection (MAS) in rice breeding during mechanical transplanting.

## Methods

### Plant materials

The genetic population for QTL mapping in this study consisted of 213 recombinant inbred lines (RILs) derived by single-seed descent from a cross between *Oryza sativa ssp. indica* cv. 9311 and *Oryza sativa ssp. japonica* cv. Nipponbare (abbreviated to NIP) [68]. The original seeds come from the State Key Laboratory of Crop Genetic Improvement, National Plant Gene Research Center, Huazhong Agricultural University, Wuhan, China. All RILs and the parents were planted at Fuyang, Hangzhou in 2017. Mature seeds were harvested and stored at 4 °C for use.

### Experimental design and seedling raising

The experiment was carried out in Fuyang, Hangzhou, China Rice Research Institute. In this study, the seedling raising method was mechanical planting technique of bowl-shaped blanket-like seedling. The substrate used for rice seedlings raising is commercial substrate in the market without adding other ingredients. The seedlings were raised on plastic nursery tray with 58 cm long, 28 cm wide and 2.5 cm height, containing 420 holes. The seeding densities were set as three levels, 2, 4 and 6 grains per hole, and called low seeding density (LD), medium seeding density (MD) and high seeding density (HD), respectively. The experiments were repeated three times on May 2018, September 2018, and May 2019, referred to as Ex1, Ex2 and Ex3, respectively. The experiment was arranged as a split-plot design. The experiment was laid out in 3 blocks of 215 main plots, each split into 3 sub-plots. The RILs and two parents were applied to the main plots and the seeding density conditions to the sub-plots. According to the experiment design, plump seeds of each line were selected to detect germination rate, and then the qualified seeds were soaking with 25% prochloraz agent. After 2 days, the seeds were transferred to greenhouse incubator to accelerate germination for 12 h. The corresponding amounts of germination grains were measured for each seedling tray, and then evenly distributed on the surface of the nursery tray by the rice precision seeder, with equal amount of substrate on the back cover. Conventional field seedling management was adopted for seedling management.

### Phenotyping of seedling traits

After 20 days of growth, the seedlings along with substrates for each genotype and seeding density in area of 10 cm × 10 cm in the middle of the nursery tray were selected and harvested. The sampling was repeated three times for each sub-plot. The phenotypes measured including seedling height (SH, cm), first leaf sheath length (FLSL, cm), first leaf length (FLL, cm), second leaf length (SLL, cm), root dry weight (RDW, mg) and shoot dry weight (SDW, mg). To determine RDW and SDW, the seedlings with substrate were dug out, placed in a sieve, and gently rinsed until no substrate remains. The average of the thirty seedlings with uniform size was treated as trait value in each sub-plot. The roots and shoots were then separated by cutting from the basal part of shoots. All the other traits were measured in the laboratory immediately following harvest, except shoot and root dry weight were measured after oven drying at 70 °C for 3 days until constant weight.

### Statistical analysis

A linear mixed model was used to estimate mean of each line, with lines as fixed effects, replications and blocks as random effects and in CropStat (v7.2.2007.3) (http://bbi.irri.org/products). The phenotypes and correlation coefficients among the traits were analyzed using R Statistics (R version 3.6.1) [69]. Analyses of variance (ANOVA) were used to test the interactions of genotype × environment (G × E) among three seedling densities. in each experiment. Broad-sense heritability (*H*) was calculated by an ANOVA using the formula:

$$ \boldsymbol{H}={\boldsymbol{\sigma}}_{\boldsymbol{G}}^{\mathbf{2}}/\left({\boldsymbol{\sigma}}_{\boldsymbol{G}}^{\mathbf{2}}+{\boldsymbol{\sigma}}_{\boldsymbol{G}\boldsymbol{E}}^{\mathbf{2}}/\boldsymbol{n}+{\boldsymbol{\sigma}}_{\boldsymbol{\varepsilon}}^{\mathbf{2}}/\boldsymbol{rn}\right) $$

where ${\sigma}_G^2$, ${\sigma}_{GE}^2$ and ${\sigma}_{\varepsilon}^2$ were the genotypic variance, genotype × environment interaction variance and residual error variance, respectively, and r was the number of replicates and n was the number of experiments.

The single-locus additive effect of each bin across the whole genome for each experiment was calculated by the half of the difference between the means of the two homozygotes ‘9311’ and ‘NIP’. Any bin with heterozygous genotype was treated as a missing value. Analysis of variance with a threshold of *F*-value of 3.89 (*P* ≤ 0.05) were then used to test the significance of the additive effect at each bin. The bins with significant additive effects were then confirmed with 1000 permutation tests. Those bins were regarded as significant at *P* ≤ 0.05, unless no more than 5% of the random *F*-values were larger than the *F*-value from the original data [54]. Two-locus epistasis for each seeding density using all possible two-locus (bin) combinations in the RIL population were resolved using two-way ANOVA with a threshold of *F*-value of 11.13 (*P* ≤ 0.001). The calculation was based on unweighted cell means and the sums of squares were multiplied by the harmonic means of the cell sizes to form the test criteria [70]. Those significant digenic interactions were then confirmed by 10,000 random permutation tests, and the resulting 10,000 *F*-values were compared with the original *F*- value. If no more than one *F*-value from the random permutations was larger than the original *F*-value, the digenic interaction was regarded as significant (*P* ≤ 0.0001) [54, 71, 72]. Since the RIL populations were homozygous, the epistatic effects were characterized as additive by additive interaction.

### QTL analysis

**Fig. 6:**
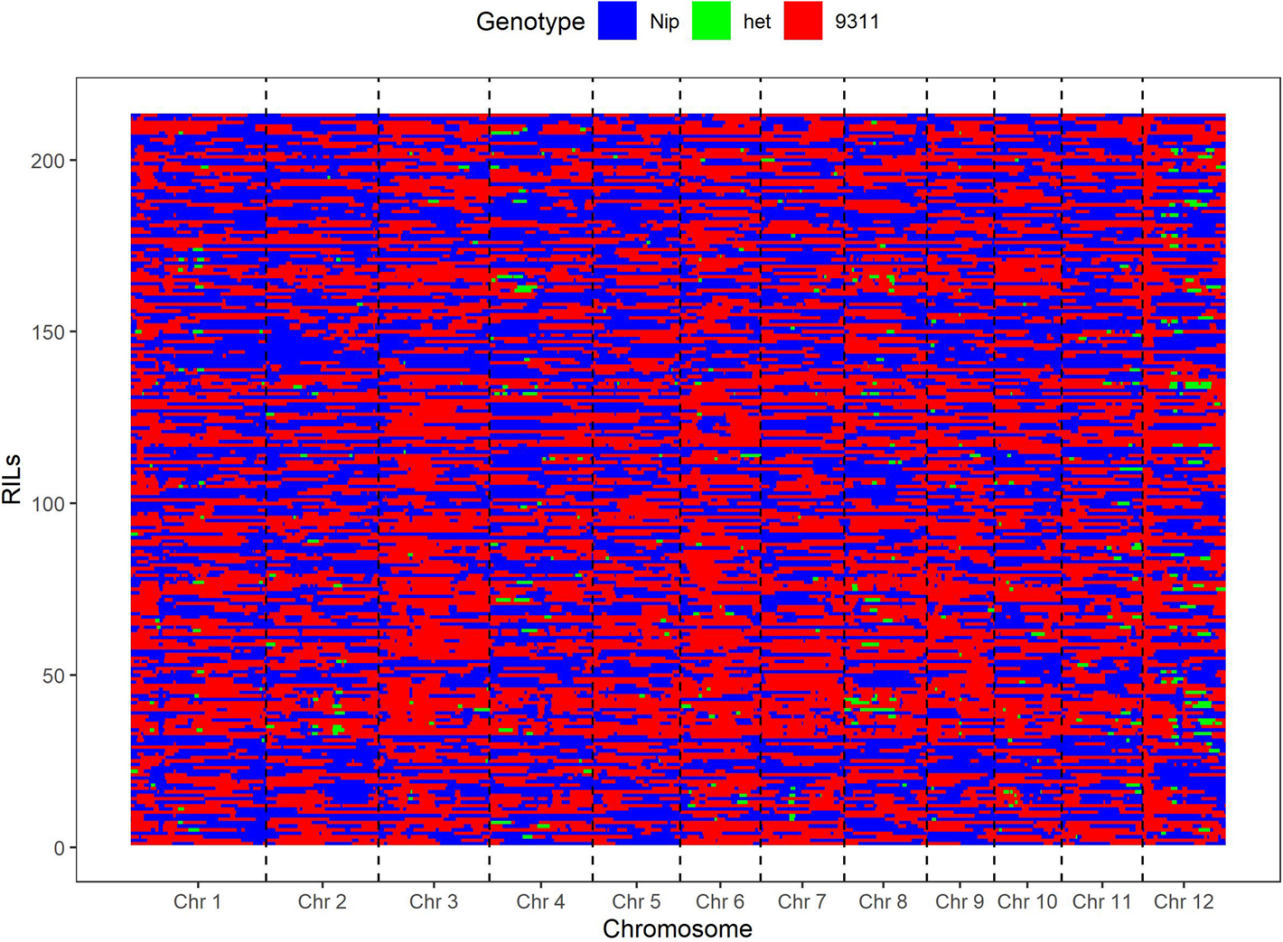
Genetic map of 2778 recombination bins for the 213 RILs. Red, 9311 genotype; blue, ‘Nipponbare’ genotype; green, heterozygous genotype

The complete linkage map of the RIL population constructed through high-density SNP marker analysis had been previously reported [68, 73]. In our study, the RILs were genotyped based on SNPs generated from the whole-genome resequencing. The recombination maps of the RILs were aligned and compared for their genotypes for a 100-kb interval, resulting in a high-density bin map consisting of 2778 bins [73]. Each bin was then treated as a genetic marker for linkage map construction using MAPMAKER/EXP version 3.0b [74], resulting in a genetic linkage map of 1564.4 cM in length, covering all 12 chromosomes (Fig. 6 and Additional file 1: Table S1). QTL analysis was performed with the composite interval mapping (CIM) procedure of R/qtl function *cim* [75]. For the high-density bin map, because the bins were different from the traditional molecular markers, the scan window of R/qtl function *cim* was set to zero [76]. The likelihood ratio statistic was computed for each bin. Significant (*P* ≤ 0.05) LOD thresholds were determined by 1000 permutation tests. One QTL whose LOD value and the percentage of phenotypic variation explained exceeded 3.0 and 10% was declared as a main-effect QTL, and otherwise a minor QTL. The location of each QTL was determined based on the LOD peak location and a 1.5 LOD-drop support interval was calculated for each QTL to obtain a 95% confidence interval [76]. The additive effect and proportion of the phenotype variance explained by each QTL were determined by the linear model using the R [69].

## Supplementary Information

**Additional file 1:** **Table S1.**Genotypes for all 2778 bins for the 213 RILs from the 9311/Nipponbare cross based on high quality SNPs. AA, Nipponbare genotype; BB, 9311 genotype; AB, heterozygous genotype. The position represents the physical position of each bin. **Table S2.** Phenotypic variation of the RIL population of 9311/Nipponbare cross among three seeding densities. SH, seedling height (cm); FLSL, first leaf sheath length (cm); FLL, first leaf length (cm); SLL, second leaf length (cm); SDW, shoot dry weight (mg); RDW, root dry weight (mg). **Table S3.** Correlation analyses among different seedling traits. LD, low density; MD, medium density; HD, high density; Ex1, Ex2 and Ex3 indicated the three experiments, respectively. SH, seedling height; FLSL, first leaf sheath length; FLL, first leaf length; SLL, second leaf length; SDW, shoot dry weight; RDW, root dry weight. * and ** indicated significant at *P* ≤ 0.05 and *P* ≤ 0.01, respectively. **Table S4.** The QTLs identified for seedling traits from the RIL population of 9311/Nipponbare cross under three seeding densities. LD, low density; MD, medium density; HD, high density; SH, seedling height; FLSL, first leaf sheath length; FLL, first leaf length; SLL, second leaf length; SDW, shoot dry weight; RDW, root dry weight. The position is the lod peak of each QTL. The interval is 1.5-LOD support interval of the QTL. Positive and negative additive effect values indicate that the allele from NIP and 9311 increase trait values, respectively. The Var is variation (%) explained by each QTL. **Table S5.** Additive effects of each bin under three seeding densities. A_score, additive effect, in which positive values indicate that alleles from NIP are in the direction of increasing the trait scores, and negative values indicate that alleles from 9311 are in the direction of increasing the score. NA, NIP and 9311 indicated the additive effect non-significant, positive significant and negative significant, respectively. LD, low density; MD, medium density; HD, high density; SH, seedling height; FLSL, first leaf sheath length; FLL, first leaf length; SLL, second leaf length; SDW, shoot dry weight; RDW, root dry weight. **Table S6.** Two-locus combinations showing significant epistatic effects under three seeding densities. LD, low density; MD, medium density; HD, high density; SH, seedling height; FLSL, first leaf sheath length; FLL, first leaf length; SLL, second leaf length; SDW, shoot dry weight; RDW, root dry weight. Var, the percentage (%) of variation explained by the interaction.

## Data Availability

All data generated or analysed during this study are included in this published article and its supplementary information files.

## Abbreviations

RILs: Recombinant inbred lines
NIP: Nipponbare
LD: Low seeding density
MD: Medium seeding density
HD: High seeding density
SH: Seedling height
FLSL: First leaf sheath length
FLL: First leaf length
SLL: Second leaf length
SDW: Shoot dry weight
RDW: Root dry weight
