# Supplementary material for: Genetic analysis of rice seedling traits related to machine transplanting under different seeding densities
Source: BMC Genet. 2020 Nov 26;21:133. doi: 10.1186/s12863-020-00952-1 (PMC7690112; doi:10.1186/s12863-020-00952-1)
Supplement: Supplementary file 1 — Additional file 1: Table S1.Genotypes for all 2778 bins for the 213 RILs from the 9311/Nipponbare cross based on high quality SNPs. AA, Nipponbare genotype; BB, 9311 genotype; AB, heterozygous genotype. The position represents the physical position of each bin. Table S2. Phenotypic variation of the RIL population of 9311/Nipponbare cross among three seeding densities. SH, seedling height (cm); FLSL, first leaf sheath length (cm); FLL, first leaf length (cm); SLL, second leaf length (cm); SDW, shoot dry weight (mg); RDW, root dry weight (mg). Table S3. Correlation analyses among different seedling traits. LD, low density; MD, medium density; HD, high density; Ex1, Ex2 and Ex3 indicated the three experiments, respectively. SH, seedling height; FLSL, first leaf sheath length; FLL, first leaf length; SLL, second leaf length; SDW, shoot dry weight; RDW, root dry weight. * and ** indicated significant at P ≤ 0.05 and P ≤ 0.01, respectively. Table S4. The QTLs identified for seedling traits from the RIL population of 9311/Nipponbare cross under three seeding densities. LD, low density; MD, medium density; HD, high density; SH, seedling height; FLSL, first leaf sheath length; FLL, first leaf length; SLL, second leaf length; SDW, shoot dry weight; RDW, root dry weight. The position is the lod peak of each QTL. The interval is 1.5-LOD support interval of the QTL. Positive and negative additive effect values indicate that the allele from NIP and 9311 increase trait values, respectively. The Var is variation (%) explained by each QTL. Table S5. Additive effects of each bin under three seeding densities. A_score, additive effect, in which positive values indicate that alleles from NIP are in the direction of increasing the trait scores, and negative values indicate that alleles from 9311 are in the direction of increasing the score. NA, NIP and 9311 indicated the additive effect non-significant, positive significant and negative significant, respectively. LD, low density; MD, me [file 12863_2020_952_MOESM1_ESM.zip › Additional file 1.docx]

**Genetic analysis of rice seedling traits related to machine transplanting under different seeding densities**

**Dan Zhu, Yuping Zhang, Jing Xiang, Yaliang Wang, Defeng Zhu, Yikai Zhang^*^ and Huizhe Chen^*^**

State Key Laboratory of Rice Biology, China National Rice Research Institute, Hangzhou, China

*** Correspondence:**Huizhe Chen: chenhuizhe@163.com

**Additional file 1**

**Table S1. Genotypes for all 2778 bins for the 213 RILs from the 9311/Nipponbare cross based on high quality SNPs.** AA, Nipponbare genotype; BB, 9311 genotype; AB, heterozygous genotype. The position represents the physical position of each bin.

**Table S2. Phenotypic variation of the RIL population of 9311/Nipponbare cross among three seeding densities**. SH, seedling height (cm); FLSL, first leaf sheath length (cm); FLL, first leaf length (cm); SLL, second leaf length (cm); SDW, shoot dry weight (mg); RDW, root dry weight (mg).

**Table S3. Correlation analyses among different seedling traits.** LD, low density; MD, medium density; HD, high density; Ex1, Ex2 and Ex3 indicated the three experiments, respectively. SH, seedling height; FLSL, first leaf sheath length; FLL, first leaf length; SLL, second leaf length; SDW, shoot dry weight; RDW, root dry weight. * and ** indicated significant at *P* ≤ 0.05 and *P* ≤ 0.01, respectively.

**Table S4. The QTLs identified for seedling traits from the RIL population of 9311/Nipponbare cross under three seeding densities.** LD, low density; MD, medium density; HD, high density; SH, seedling height; FLSL, first leaf sheath length; FLL, first leaf length; SLL, second leaf length; SDW, shoot dry weight; RDW, root dry weight. The position is the lod peak of each QTL. The interval is 1.5-LOD support interval of the QTL. Positive and negative additive effect values indicate that the allele from NIP and 9311 increase trait values, respectively. The Var is variation (%) explained by each QTL.

**Table S5. Additive effects of each bin under three seeding densities.** A_score, additive effect, in which positive values indicate that alleles from NIP are in the direction of increasing the trait scores, and negative values indicate that alleles from 9311 are in the direction of increasing the score. NA, NIP and 9311 indicated the additive effect non-significant, positive significant and negative significant, respectively. LD, low density; MD, medium density; HD, high density; SH, seedling height; FLSL, first leaf sheath length; FLL, first leaf length; SLL, second leaf length; SDW, shoot dry weight; RDW, root dry weight.

**Table S6. Two-locus combinations showing significant epistatic effects under three seeding densities.** LD, low density; MD, medium density; HD, high density; SH, seedling height; FLSL, first leaf sheath length; FLL, first leaf length; SLL, second leaf length; SDW, shoot dry weight; RDW, root dry weight. Var, the percentage (%) of variation explained by the interaction.
